# Boron neutron capture therapy induces apoptosis of glioma cells through Bcl-2/Bax

**DOI:** 10.1186/1471-2407-10-661

**Published:** 2010-12-02

**Authors:** Peng Wang, Haining Zhen, Xinbiao Jiang, Wei Zhang, Xin Cheng, Geng Guo, Xinggang Mao, Xiang Zhang

**Affiliations:** 1 Department of Neurosurgery of Xijing Hospital, Fourth Military Medical University, Xi'an, Shaanxi 710032, P.R.China; 2 Northwest Institute of Nuclear Technology, Xi'an, Shaanxi 710024, PR China; 3 Department of Biochemistry and Molecular Biology, Fourth Military Medical University, Xi'an, Shaanxi 710032, PR China

## Abstract

**Background:**

Boron neutron capture therapy (BNCT) is an alternative treatment modality for patients with glioma. The aim of this study was to determine whether induction of apoptosis contributes to the main therapeutic efficacy of BNCT and to compare the relative biological effect (RBE) of BNCT, γ-ray and reactor neutron irradiation.

**Methods:**

The neutron beam was obtained from the Xi'an Pulsed Reactor (XAPR) and γ-rays were obtained from [^60^Co] γ source of the Fourth Military Medical University (FMMU) in China. Human glioma cells (the U87, U251, and SHG44 cell lines) were irradiated by neutron beams at the XAPR or [^60^Co] γ-rays at the FMMU with different protocols: Group A included control nonirradiated cells; Group B included cells treated with 4 Gy of [^60^Co] γ-rays; Group C included cells treated with 8 Gy of [^60^Co] γ-rays; Group D included cells treated with 4 Gy BPA (p-borono-phenylalanine)-BNCT; Group E included cells treated with 8 Gy BPA-BNCT; Group F included cells irradiated in the reactor for the same treatment period as used for Group D; Group G included cells irradiated in the reactor for the same treatment period as used for Group E; Group H included cells irradiated with 4 Gy in the reactor; and Group I included cells irradiated with 8 Gy in the reactor. Cell survival was determined using the 3-(4,5-dimethylthiazol-2-yl-2,5-diphenyltetrazolium (MTT) cytotoxicity assay. The morphology of cells was detected by Hoechst33342 staining and transmission electron microscope (TEM). The apoptosis rate was detected by flow cytometer (FCM). The level of Bcl-2 and Bax protein was measured by western blot analysis.

**Results:**

Proliferation of U87, U251, and SHG44 cells was much more strongly inhibited by BPA-BNCT than by irradiation with [^60^Co] γ-rays (*P *< 0.01). Nuclear condensation was determined using both a fluorescence technique and electron microscopy in all cell lines treated with BPA-BNCT. Furthermore, the cellular apoptotic rates in Group D and Group E treated with BPA-BNCT were significantly higher than those in Group B and Group C irradiated by [^60^Co] γ-rays (*P *< 0.01). The clonogenicity of glioma cells was reduced by BPA-BNCT compared with cells treated in the reactor (Group F, G, H, I), and with the control cells (*P *< 0.01). Upon BPA-BNCT treatment, the Bax level increased in glioma cells, whereas Bcl-2 expression decreased.

**Conclusions:**

Compared with γ-ray and reactor neutron irradiation, a higher RBE can be achieved upon treatment of glioma cells with BNCT. Glioma cell apoptosis induced by BNCT may be related to activation of Bax and downregulation of Bcl-2.

## Background

Glioma is one of the most aggressive human malignancies. Despite aggressive surgery combined with adjuvant radiotherapy and chemotherapy, patient prognosis remains poor. It is thus essential to develop novel therapeutic strategies to treat glioma. Boron neutron capture therapy (BNCT) is a highly selective treatment modality that can target the tumor without causing excessive radiation damage to the normal tissues [1]. The ability of [^10^B] to capture thermal neutrons, and to then disintegrate immediately into a He nucleus (an α particle) and a Li nucleus, is utilized in BNCT [2]. Both the He and Li nuclei have high lineal energy transfer (LET) values, with path-lengths approximately the diameter of a single cell; the lethality of these nuclei is thus primarily limited to boron-containing cells [3]. BNCT has been carried out in numerous studies using syngeneic rat gliomas [3-6]. Approaches such as administering [^10^B] by intra-arterial injection with or without blood-brain barrier (BBB) disruption [7-9], or intracerebral delivery of high molecular weight agents by means of convection enhanced delivery following neutron beam irradiation [2,10], confirm that BNCT is highly effective for treatment glioma xenograft models. In clinic, BNCT has been used in various countries to treat patients with high-grade glioma, melanoma, liver metastasis of colon adenocarcioma, malignant melanomas, oral cancer, and therapeutically refractory, recurrent tumors of the head and neck [3,6,11-15]; while the mechanism of BNCT-induced cell death remains unclear.

We treated U87, U251, and SHG44 glioma cells with BNCT *in vitro*, using L-p-borono-phenylalanine (L-BPA) as the boron carrier and the thermal neutron source of the Xi'an Pulsed Reactor (XAPR) in China as the irradiator. The relative biological effect (RBE) of BNCT on tested cells, especially with respect to apoptosis induction, was assessed, and a possible mechanism of BNCT action was presented.

## Methods

### Glioma cell culture

The human glioblastoma multiforme cell lines U87 and U251 were obtained from the American Type Culture Collection (ATCC; Manassas, VA). The human anaplastic astrocytoma cell line SHG44 was purchased from the Institute of Biochemistry and Cell Biology (IBCB; Shanghai, China). Cells were cultured in Dulbecco's modified Eagle's medium (DMEM) (Gibco, Grand Island, NY) with 10% (v/v) fetal bovine serum (Gibco) and 0.5% (w/v) penicillin-streptomycin solution (Gibco), at 37°C under 5% CO_2 _and 95% air (both v/v).

### Boron uptake by glioma cells *in vitro*

[^10^B]-enriched L-BPA (Ryscor Inc., Raleigh, NC) was used in the study. L-BPA was added to the culture media of glioma cells to 50 μg [^10^B]/ml. Cells in the exponential phase of growth were cultured in boron-containing medium for 3, 6, 12, or 24 h before harvesting, and boron concentrations were determined by inductively coupled plasma atomic emission spectroscopy (ICP-AES) (VISTA-MPX; Varian Co., Walnut Creek, CA) according to a previously described method [16].

### *In vitro *irradiation

*In vitro *irradiation of the three cell lines was performed using the neutron beam at the XAPR whereas *in vitro *γ-ray irradiation by the [^60^Co] γ source was performed in the Fourth Military Medical University (Xi'an, China). Cells were divided into nine groups. Group A was control nonirradiated cells; Group B cells were irradiated with 4 Gy of [^60^Co] γ-rays; Group C cells were irradiated with 8 Gy of [^60^Co] γ-rays; Group D cells were irradiated with 4 Gy BPA-BNCT; Group E cells were irradiated with 8 Gy BPA-BNCT; Group F cells were irradiated in the XAPR for the same treatment period as used for Group D; Group G cells were irradiated in the XAPR for the same treatment period as used for Group E; Group H cells were irradiated with 4 Gy in the XAPR; and Group I cells were irradiated with 8 Gy in the XAPR. In Group D and Group E with BPA-BNCT, cells were incubated for 24 h in medium with BPA and washed three times with phosphate-buffered saline (PBS); the medium was next replaced with boron-free medium; cells were exposed to the XAPR with irradiation doses of 4 Gy and 8 Gy, respectively. In the γ-ray-treated Group B and Group C, cells incubated in growth medium without BPA were exposed to the γ-rays with irradiation doses of 4 Gy and 8 Gy, respectively. In Group F and Group G, cells incubated in medium without BPA were exposed to the XAPR with identical treatment durations of Group D and Group E, respectively. In Group H and Group I, cells incubated in growth medium without BPA were exposed to the XAPR with irradiation doses of 4 Gy and 8 Gy, respectively. Irradiated and nonirradiated cells were placed at room temperature and under a normal atmosphere during irradiation. At the beginning of the experiment, all cells were in the exponential phase of growth and at a density of 3×10^5^/ml. The components of the reactor neutron source and the [^60^Co] γ source were shown in Table 1; the irradiation periods of various groups were listed in Table 2; the dose components of BNCT were listed in Table 3 and Table 4. The irradiation doses were calculated according to previous studies [17,18].

**Table 1:** Dose rates for reactor neutron source and [60Co] γ source

Components		Dose rate (Gy/min)
Dose rates for the reactor neutron source	Thermal neutron	1.04×10^-3^
	Epithermal neutron	2.48×10^-4^
	Fast neutron	1.07×10^-1^
	γ-ray	1.57×10^-2^
	^10^B(n,ɑ)^7^Li	5.09×10^-4^/ppm^10^B

Dose rate for the [^60^Co] γ source		1.40×10^1^

**Table 2:** The irradiation periods of various groups

	Group A(min)	Group B(min)	Group C(min)	Group D(min)	Group E(min)	Group F(min)	Group G(min)	Group H(min)	Group I(min)
U87	64	0.29	0.57	27	54	27	54	32	64
U251	64	0.29	0.57	25	50	25	50	32	64
SHG44	64	0.29	0.57	28	56	28	56	32	64

**Table 3:** The dose components of BNCT 4 Gy

	Thermal neutron (Gy)	Epithermal neutron (Gy)	Fast neutron (Gy)	γ-ray (Gy)	^10^B(n,ɑ)^7^Li (Gy)
U87	0.028	0.007	2.889	0.423	0.653
U251	0.026	0.007	2.675	0.392	0.900
SHG44	0.029	0.007	2.996	0.439	0.529

**Table 4:** The dose components of BNCT 8 Gy

	Thermal neutron (Gy)	Epithermal neutron (Gy)	Fast neutron (Gy)	γ-ray (Gy)	^10^B(n,ɑ)^7^Li (Gy)
U87	0.056	0.013	5.778	0.846	1.306
U251	0.052	0.013	5.350	0.748	1.800
SHG44	0.058	0.013	5.992	0.878	1.058

### Proliferation assays

Cell survival was determined using the 3-(4,5-dimethylthiazol-2-yl-2,5-diphenyltetrazolium (MTT) (Sigma, St. Louis, MO) assay. After irradiation, cells were trypsinized and washed, and cell concentrations were adjusted to 10^4 ^cells/ml. Cells were seeded into the wells of 96-well microplates (Nunc, Roskilde, Denmark) and incubated for 2, 4, or 6 days. The culture medium was removed and replaced with 100 μl aliquots of fresh medium, without serum, containing 0.5 mg/ml MTT, followed by 4 h of incubation at 37°C. Next, medium was aspirated from the wells and 100 μl dimethyl sulfoxide (DMSO) was added to each well, to dissolve formazan crystals. Optical density (OD) was measured using an enzyme-linked immunosorbent method. Cell numbers were derived from OD values by reference to a standard cell number-OD curve.

### Hoechst staining

At 12 h, 24 h, 36 h, and 48 h after treatment with BPA-BNCT, cells were fixed for 10 min in 4% (v/v) paraformaldehyde, and then incubated with Hoechst 33342 dye (Sigma) (10 μg/ml) for 10 min. After washed with PBS, cells were observed using an inverted fluorescence microscope (IX70; Olympus, Tokyo, Japan).

### Electron microscopy

Forty-eight hours after irradiation, cells were detached from plates, washed, suspended in PBS, and concentrated by centrifugation. Cell samples were fixed in 2.5% (v/v) glutaraldehyde in 0.1 M phosphate buffer (pH 7.4), postfixed in 2% (w/v) buffered osmium tetroxide for 2 h, and dehydrated in ethanol. Specimens for transmission electron microscopy were embedded in Epon. Thin sections were cut using an ultramicrotome and double-stained with uranyl acetate and lead citrate. Electron micrography was performed (JEM-2000EX; JEOL, Tokyo, Japan) using an operating voltage of 80 kV.

### AnnexinV/PI analysis

Twelve hours, 24 h, 36 h, and 48 h after irradiation, cells were trypsinized, counted, washed twice in ice-cold PBS solution, and resuspended in 1×binding buffer (10 mM HEPES/NaOH [pH 7.4], 140 mM NaCl, and 2.5 mM CaCl_2_). Next, 5 μl of annexin V-FITC (AV) (R&D Systems Europe Ltd., Abingdon, UK) and 10 μl of propidium iodide (PI) (Sigma) were added to 100 μl of cell suspension, followed by incubation for 15 min at room temperature in the dark. Finally, 400 μl of binding buffer was added to each sample, which was held on ice prior to analysis on a FACSCalibur (Becton Dickinson Labware, Franklin Lakes, NJ) flow cytometer. Ten thousand cells per sample were analyzed.

### Clonogenic assay

After irradiation, 200 treated cells were plated in six-well flat-bottomed microplates (Nunc) and incubated at 37°C in a humidified incubator under 5% (v/v) CO_2 _for 2 weeks. The cells were fixed in methanol and stained with Giemsa's solution. Colonies ≥ 50 μm in diameter were counted [19].

### Western blot analysis

Twelve hours and 24 h after irradiation, cells were washed twice in ice-cold PBS and protein extracts of U87, U251, and SHG44 cells were prepared by lysis in RIPA buffer (150 mM NaCl, 1% [v/v] NP-40, 0.5% [w/v] sodium deoxycholate, 0.1% [w/v] sodium dodecyl sulfate (SDS), 50 mM Tris HCl [pH 8], 10 mM EDTA, and 1 mM PMSF [Sigma]) for 30 min at 4°C. Samples were next centrifuged for 15 min at 15,000 g. Protein concentrations of supernatants were determined. For each sample, 60 μg of protein was loaded on a 12.5% (w/v) SDS-polyacrylamide gel, electrophoresed, and transferred to a nitrocellulose membrane (Protran; Schleicher and Schuell, Florham Park, NJ). Each membrane was blocked for 1 h at room temperature with blocking buffer (TBS containing 0.1% [v/v] Tween 20 [Sigma] and 5% [w/v] milk powder). Primary antibodies (applied for 1 h at room temperature, or overnight at 4°C) were: anti-Bcl-2 (Santa Cruz Biotechnology, Inc., Heidelberg, Germany), anti-Bax (Santa Cruz), and anti-actin (mouse monoclonal C-2, Santa Cruz). Antibodies were diluted 1:1,000, except for the anti-actin antibody (1:500). Thereafter, membranes were incubated for 1 h with HRP-labeled secondary antibodies (Amersham Pharmacia Biotech, Uppsala, Sweden), either sheep anti-mouse (diluted 1:2,500) or donkey anti-rabbit (1:5,000), and the blots were finally developed using an ECL system, according to the manufacturer's instructions (Amersham Bioscience, Buckinghamshire, UK).

### Statistical analysis

Outcome variables are expressed as means ± standard deviations (SDs). Statistical analysis was performed using SPSS Version 13.0 (SPSS, Chicago, IL). ANOVA was used for data analysis. All statistical tests were two-sided, and *P *values < 0.05 were considered to be statistically significant.

## Results

### Boron uptake

**Figure 1:**
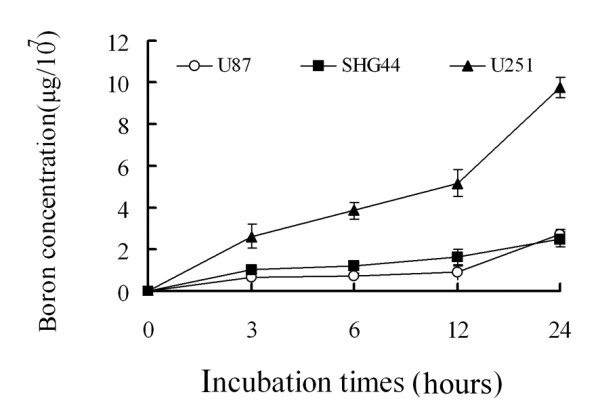
**Time course of boron accumulation**. Data represent the mean ± SD of triplicate experiments.

As expected, [^10^B] was taken up by U87, U251, and SHG44 cells during incubation for 24 hours in medium containing 50 μg [^10^B]/ml. The boron concentrations in cells eventually reached 2.72 ± 0.25 μg/10^7 ^cells, 9.78 ± 0.49 μg/10^7 ^cells, and 2.48 ± 0.34 μg/10^7 ^cells for the U87, U251, and SHG44 cell lines, respectively, at 24 h (Figure 1).

### Tumor cell numbers fall after BPA-BNCT treatment

**Figure 2:**
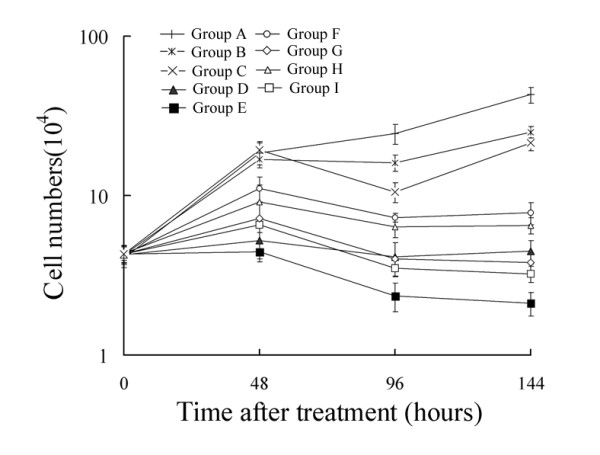
**Growth curves of U87 cells in different groups**. Group A; control nonirradiated cells; Group B: cells treated with 4 Gy of [^60^Co] γ-rays; Group C: cells treated with 8 Gy of [^60^Co] γ-rays; Group D: cells treated with 4 Gy BPA-BNCT; Group E: cells treated with 8 Gy BPA-BNCT; Group F: cells irradiated in the reactor for the same treatment period as used for Group D; Group G: cells irradiated in the reactor for the same treatment period as used for Group E; Group H: cells irradiated with 4 Gy in the reactor; Group I: cells irradiated with 8 Gy in the reactor. Data represent the mean ± SD of triplicate experiments.

The inhibitory effect of BPA-BNCT on proliferation of U87 cells was much more significant than that afforded by γ-rays, as confirmed by the MTT assay (*P *< 0.01, Figure 2). Any inhibitory effect was marginal in cells receiving 4 Gy or 8 Gy of γ-rays, whereas significant inhibition was observed in cells treated with 4 Gy or 8 Gy of BPA-BNCT. The number of viable U87 cells decreased in 48 h after treatment in all of Groups D, E, F, G, H, and I, and the most potent inhibition was observed in the group receiving 8 Gy of BPA-BNCT. Similar effects were observed in SHG44 and U251 cells (data not shown).

### BNCT induces apoptosis

**Figure 3:**
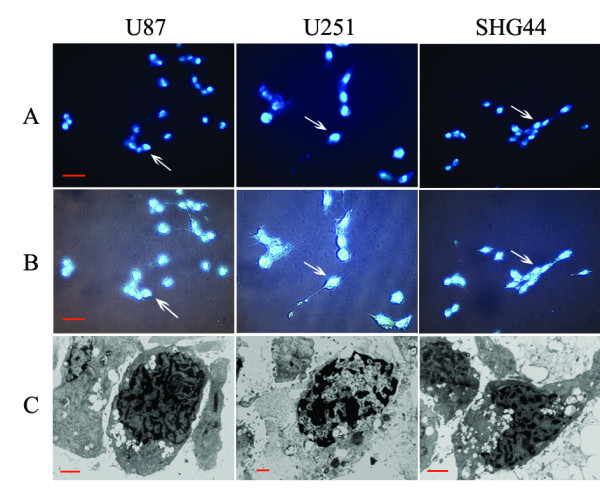
**Morphologic changes in glioma cells following BPA-BNCT treatment**. (A) Hoechst staining. White arrows indicate apoptotic cells in U87 cells, U251 cells, and SHG44 cells, respectively. Original magnification×400; bar: 10 μm. (B) Phase-contrast microscopy. White arrows indicate apoptotic cells in U87 cells, U251 cells, and SHG44 cells, respectively. Original magnification×400; bar: 10 μm. (C) Transmission electron microscopy. Left, U87 cells; original magnification×6,000; bar: 1 μm; middle, U251 cells; original magnification×5,000; bar: 1 μm; right, SHG44 cells; original magnification×6,000; bar: 1 μm.

Typical apoptotic morphological changes were found in all of the U87, U251, and SHG44 cell lines 12 h after treatment with BPA-BNCT; the changes included shrinkage, deformation, and detachment from culture dishes. Nuclear condensation and chromatin margination were evident by Hoechst 33342 staining (Figure 3A, 3B), and chromatin margination, nuclear condensation, and segmentation were noted on transmission electron microscopy (Figure 3C).

**Figure 4:**
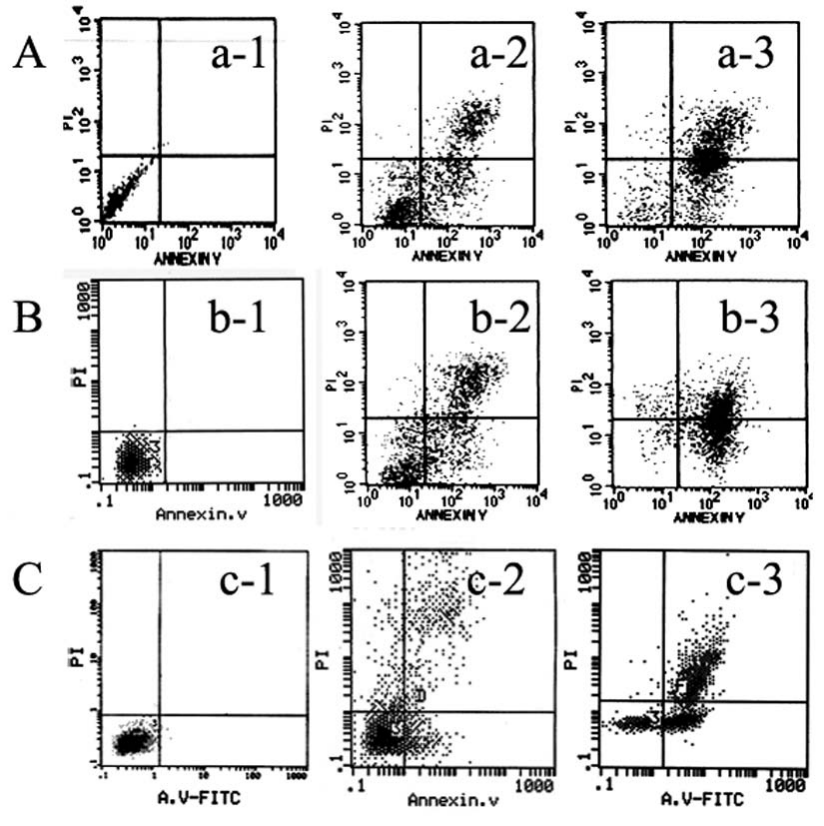
**Apoptosis of glioma cells induced by BPA-BNCT**. (A) U87 cells; a-1, nonirradiated control; a-2, 48 h after treatment with 4 Gy BPA-BNCT; a-3, 48 h after treatment with 8 Gy BPA-BNCT. (B) SHG44 cells; b-1, nonirradiated control; b-2, 48 h after treatment with 4 Gy BPA-BNCT; b-3, 48 h after treatment with 8 Gy BPA-BNCT. (C) U251 cells; c-1, nonirradiated control. c-2, 48 h after treatment with 4 Gy BPA-BNCT; c-3, 48 h after treatment with 8 Gy BPA-BNCT.

**Figure 5:**
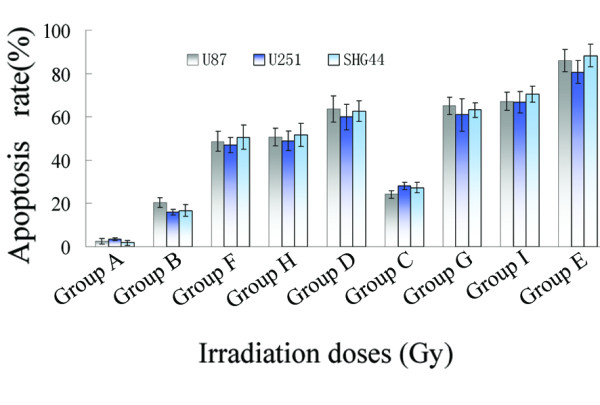
**At 48 h after irradiation the apoptotic frequencies of glioma cells in the nine groups**. Group A; control nonirradiated cells; Group B: cells treated with 4 Gy of [^60^Co] γ-rays; Group C: cells treated with 8 Gy of [^60^Co] γ-rays; Group D: cells treated with 4 Gy BPA-BNCT; Group E: cells treated with 8 Gy BPA-BNCT; Group F: cells irradiated in the reactor for the same treatment period as used for Group D; Group G: cells irradiated in the reactor for the same treatment period as used for Group E; Group H: cells irradiated with 4 Gy in the reactor; Group I: cells irradiated with 8 Gy in the reactor. Data represent the mean ± SD of triplicate experiments.

The apoptotic frequency of U87 cells treated with 4 Gy or 8 Gy of BPA-BNCT was significantly higher than that of cells treated with equivalent doses of γ-rays (*P *< 0.01), as shown by annexin V/PI staining (Figure 4, 5). Furthermore, the apoptotic frequency of cells treated with 8 Gy BPA-BNCT was higher than that seen after treatment with 4 Gy BPA-BNCT (*P *< 0.01). Also, the apoptotic frequencies of U87 cells after BPA-BNCT treatment were greater than those noted after delivery of equivalent reactor doses of irradiation (*P *< 0.05). Similar effects were observed in SHG44 and U251 cells (Figure 4, 5).

### BNCT inhibits colony formation

The survival rates of nonirradiated control cells were set at 100%. Cell survival proportions were shown in Table 5. Survival analysis of U87, U251, and SHG44 cells showed that all three cell lines were inhibited more potently by BNCT than by irradiation with [^60^Co] γ-rays (*P *< 0.01) or by general reactor neutron irradiation (*P *< 0.01).

**Table 5:** Survival fractions of glioma cells in different groups

Group	Survival fraction (%)		
	**U87**	**U251**	**SHG44**

Unirradiated control (A)	100	100	100
Irradiated in ^60^Co by 4 Gy (B)	68.1	63.7	64.5
Irradiated in ^60^Co by 8 Gy (C)	10.9	13.6	12.4
Irradiated in reactor for the same treatment period as used for Group D (F)	3.1	2.3	2.5
Irradiated in reactor for the same treatment period as used for Group E (G)	1.6	1.4	1.6
Irradiated in reactor by 4 Gy (H)	2.9	2.2	2.6
Irradiated in reactor by 8 Gy (I)	1.4	0.9	1.2
Irradiated in reactor with BNCT by 4 Gy (D)*	< 0.01%	< 0.01%	< 0.01%
Irradiated in reactor with BNCT by 8 Gy (E)**	< 0.01%	< 0.01%	< 0.01%

*P < 0.01 vs A, B, F and H **P < 0.01 vs A, C, G, I and D

### Altered expression of Bcl-2 and Bax after BNCT

**Figure 6:**
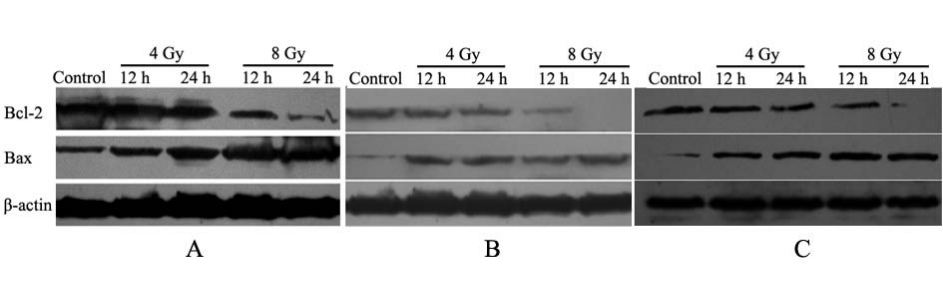
**The expression of Bcl-2 and Bax proteins in (A) U87 cells, (B) U251 cells, and (C) SHG44 cells, 12 h and 24 h after treatment with 4 Gy or 8 Gy BPA-BNCT**.

In all of U87, U251, and SHG44 cells, Bcl-2 was initially abundantly expressed, whereas Bax expression was low. After treatment with 4 Gy or 8 Gy of BPA-BNCT, Bcl-2 expression was down-regulated, while Bax expression increased simultaneously. Furthermore, 24 h after treatment with 4 Gy BPA-BNCT, Bcl-2 expression was lower than that seen 12 h after treatment with 4 Gy BPA-BNCT, and the level of Bax expression was higher. The levels of Bcl-2 and Bax expression were similar 12 h and 24 h after treatment with 8 Gy BPA-BNCT (Figure 6).

## Discussion

Two criteria must be fulfilled when BNCT is to be applied: an appropriate source of high-flux thermal neutrons is necessary, along with a boron carrier that is able to concentrate in glioma tissues. Therefore, we initially verified uptake of BPA by glioma cells *in vitro*. We found that the boron concentration in the three glioma cell lines increased constantly over the first 24 hours of incubation. Irradiation was conducted at this time, and the [^10^B] level attained the theoretical requirement, in that at least 10^9 ^[^10^B] atoms accumulated in each glioma cell and the thermal neutron fluence could attain 10^12^-10^13^n·cm^-2^, to produce a lethal discharge of high-LET particles [20].

BNCT can inhibit cell proliferation because the RBE is high, as has been shown in animal models. In the present study, although the three glioma cell lines differed in boron concentration after exposure to BPA, the therapeutic efficiency of BNCT was similar in all three lines because the RBE was high. Proliferation of glioma cells was significantly suppressed by BNCT, and the clonogenicity of glioma cells after BNCT treatment was less than 0.01% of control values.

Apoptosis, an active process of cellular suicide, is an important means of radiation-induced cell death [21], and may contribute to the therapeutic efficacy of BNCT. Many triggers of apoptosis are known, including cytogenetic alterations such as micronuclei (MN) formation and chromosomal aberrations (CA) within cellular systems [22]. Furthermore, several studies have sought to correlate the extent of apoptosis after irradiation with cellular radiosensitivity [23]. Given that BNCT works mainly through the discharge effects of high-LET Li nuclei and α particles, we investigated whether BNCT could induce apoptosis of glioma cells. Typical apoptotic morphological changes were confirmed in U87, U251, and SHG44 cells after BNCT treatment, using transmission electron microscopy and Hoechst 33342 staining. Furthermore, previous studies have shown that BPA has no toxicity effect on cells at a dosage up to 110 μg [^10^B]/ml [24]. Few apoptotic changes were apparent in nonirradiated control cells, showing, for the first time, that the pro-apoptotic effect of BNCT plays an important role in the reduction of cell proliferation. Masunaga et al. described that BNCT induced apoptosis *in vivo*, the proportion of apoptotic cells increased slightly 6 h after BNCT, and the apoptotic rate was relatively low [17,18]. Fujita et al. stated that apoptosis was a form of cell death induced by BNCT [25]. While Aromando et al. reported that apoptosis did not have a significant role in BNCT-induced hamster cheek pouch tumor control [26], and Kamida et al. described that differences in apoptotic cells pre- and post-BNCT in human oral squamous cell carcinoma xenografts were minimal [27]. Whether BNCT can induce significant apoptosis rate in glioma cells *in vivo *and whether the pro-apoptotic effect is cell-type-dependant deserved further study.

It is well established in mammalian systems that high-LET irradiations have more pronounced effects on biological systems than that of low LET [22], owing to the characteristic mode of energy deposition along a track. Such energy generates complex forms of radiation damage to biological molecules. The transfer of the energy of high-LET radiation to such molecules can break chemical bonds and ionize various cellular components.

In the present study, AnnexinV/PI analysis not only confirmed the pro-apoptotic effect of BNCT, but also showed that apoptosis frequencies were higher in groups treated with BNCT compared with γ-rays. Moreover, the apoptotic frequencies in BPA-BNCT groups were higher than those of the irradiated controls. Additionally, when the absorbed doses were identical, both the proliferation and the clonogenicity of glioma cells were suppressed by BNCT to an extent significantly greater than seen after exposure to a neutron beam. Thus, the RBE of BNCT is higher than that of either a neutron beam or γ-rays. In the present study, the contribution of a boron-dependent dose to the total BNCT dose was low. Thus, with optimization of the reactor and improvement in the boron-dependent dose, the RBE of BNCT can be further increased.

Previous studies have shown that the intrinsic apoptotic pathway (involving the mitochondria) plays a key role in the regulation of apoptosis in response to irradiation [28]. Bcl-2 family proteins interact to permeabilize the mitochondrial membrane, thus playing an important role in irradiation-induced apoptosis [29-31]. Bcl-2 and Bax, the two main members of the Bcl-2 protein family, function as tumor anti-apoptotic and pro-apoptotic factors, respectively [32-34]. Bax can cause release of cytochrome c after formation of the hetero-oligomer Bax/Bak, whereas Bcl-2 can engage with activator proteins or Bax/Bak, thereby sequestering these proteins [28]. We detected expression of Bcl-2 and Bax in all of U87, U251, and SHG44 cells after BNCT treatment in efforts to understand the impact of BNCT on the mitochondrial pathway of apoptosis. Down-regulation of Bcl-2 and up-regulation of Bax synthesis after BNCT showed that apoptosis induced by BNCT may be mediated through the mitochondrial pathway.

A Bcl-2 antisense has been developed as a gene therapeutic agent to treat cancer, such as: melanoma carcinoma, multiple myeloma, and small cell lung cancer [28]. Elimination of Bcl-2 induced by BNCT indicates that a directed Bcl-2-targeting strategy may be combined with BNCT, thus possibly leading to synergistic reduction of Bcl-2; this possibility deserves further investigation.

Our investigation of BPA-BNCT therapy was conducted *in vitro*, and many constraints are involved in attempts to extrapolate *in vitro *data to the *in vivo *context. During BNCT, the normal brain tissue and tumors are non-specifically irradiated by reactor-generated mixed irradiation beam components, and the patient tolerance to BNCT could be impaired. When it is considered that the RBE of BNCT is higher than that of γ-rays, neutron beam, and X-rays, all of which are widely used as treatment options for patients with brain tumors, the absorbed dose from BNCT can be much lower than those strategies. This will limit irradiation damage to normal tissue. The boron level in blood and brain parenchyma also contributes to the irradiation dose to normal tissue. With the development of boron containing agents and the optimization of strategies of delivery, [^10^B] can be highly accumulated in tumor cells and the level in normal cells becomes relatively lower [2,13,35,36]. Moreover, BNCT relies mainly on the use of boron compounds, and not on a neutron beam *per se*, to selectively irradiate a tumor [37]. Thus, the optimization of selective boron delivery to tumor cells is an important area of BNCT research. As known the BBB can impede the uptake of boron-containing compounds by tumor cells, with the invasive properties of glioma tumors, the BBB can be destroyed and the boron uptake by tumor cells could be increased. Drugs such as mannitol that transiently compromise BBB integrity have been used to improve boron absorbance by tumor cells. Overall, the use of BNCT *in vivo *will likely become effective, with a reduction in side effects and improvements in tumor-targeting boron compounds, following further development.

## Conclusions

Altogether, induction of apoptosis contributes to the main therapeutic efficacy of BNCT to glioma cells *in vitro*. Activation of Bax and downregulation of Bcl-2 are involvement in the apoptosis triggered by BNCT. Moreover, the RBE of BPA-BNCT is higher than that of γ-ray and reactor neutron irradiation. Understanding the mechanisms involved in BNCT will ultimately contribute to the enhancement of the therapeutic effectiveness of this therapeutic modality.

## Competing interests

The authors declare that they have no competing interests.

## Authors' contributions

Peng Wang, Haining Zhen, and Xinbiao Jiang carried out the cell culture and experiment of irradiation. Peng Wang and Xin Cheng carried out Proliferation assays, Hoechst staining, Electron microscopy assay, AnnexinV/PI analysis, Colony formation assay, and drafted the manuscript. Wei Zhang, Geng Guo, and Xinggang Mao participated in the design of the study and performed the statistical analysis. Xiang Zhang conceived of the study, and participated in its design and coordination. All authors read and approved the final manuscript.

## Pre-publication history

The pre-publication history for this paper can be accessed here:

http://www.biomedcentral.com/1471-2407/10/661/prepub
